# Processing abstract concepts through interaction: effects on social recognition

**DOI:** 10.1007/s10339-026-01388-8

**Published:** 2026-08-18

**Authors:** Mihai Alexandru Bîclea, Ana Cosmoiu

**Affiliations:** 1 Faculty of Sociology and Social Work, University of Bucharest, Bucharest, Romania; 2 Department of Psychology and Cognitive Science, University of Bucharest, Bucharest, Romania

**Keywords:** Abstract concepts, Recognition memory, Social stimuli

## Abstract

Building on the importance of sociality in grounding abstract concepts, this study investigated how concept type shapes recognition memory for social stimuli. Participants engaged in text-based dialogues about abstract or concrete concepts with virtual agents, after which recognition of names and faces, as well as perceived closeness, was measured. The procedure included a preliminary, context-based validation of linguistic dimensions adapted from Italian into Romanian, given the absence of prior norming studies. Discussions of abstract concepts elicited more uncertainty expressions, questions, turn-taking, general statements, affective content, and overt beliefs, thus offering converging evidence for the adequacy of the translated stimuli and previously reported differences in socio-cognitive engagement. Differential recognition effects facilitated by abstract concept conversations were observed for faces, but not for names. The higher salience of faces could not be ascribed straightforwardly to cognitive load or faster processing, as this interpretation was incompatible with the reaction times observed, which did not differ across conditions in either the encoding or the recognition phase. This, coupled with the absence of corresponding effects for names, which are typically recognized at least as well as faces in matched recognition paradigms, is consistent with a greater involvement of social cognitive processes during abstract concept processing. Overall, the results indicate that abstract concept processing modulates recognition memory for social stimuli. The experimental design is intended to be simple and reproducible, enabling cumulative future research on the relationship between concept types and other cognitive processes.

## Introduction

Human language rests on a fundamental asymmetry: while concrete words (e.g., chair, apple) refer to entities directly accessible to perception and action, abstract words (e.g., justice, love, freedom) denote concepts whose referents cannot be directly experienced through the senses. Embodied theories of cognition (Barsalou 1999, 2012; Borghi 2005; Gallese and Lakoff 2005) propose that conceptual knowledge is grounded in sensorimotor simulations of perceptual and motor experience, an account that has proven powerful for concrete concepts, but faces a well-known challenge when applied to abstract ones. The problem of grounding abstract concepts (Dove 2021; for a review, see Borghi et al. 2017) refers to the explanatory gap between sensorimotor, perceptual, action, and affective systems, on the one hand, and highly abstract concepts that lack direct perceptual referents, on the other.

Multirepresentational approaches, most notably the Words as Social Tools (WAT) theory, address this challenge by supplementing sensorimotor grounding with additional grounding sources of meaning, including interoceptive, emotional, linguistic, and social experiences (Borghi and Binkofski 2014; Borghi et al. 2019). The present study investigates one implication of the greater extent of social and linguistic grounding of abstract concepts: whether discussing abstract, compared to concrete, concepts in a simulated computer-mediated, text-based dialogue modulates memory for the social identity of interlocutors and perceived psychological closeness. In doing so, the study replicates Villani et al.’s (2022) conversational paradigm in a Romanian sample and extends it by linking abstract concept discussion to a social memory mechanism.

## Abstract concepts and social grounding in dialogue

According to WAT, both abstract and concrete concepts re-enact prior multimodal experiences with their referents (Borghi and Cimatti 2009; Borghi and Binkofski 2014; Borghi et al. 2019; Mazzuca et al. 2025b). Abstract concepts rely more heavily on linguistic, interoceptive, and social networks, whereas concrete concepts rely relatively more heavily on perceptual and action-related experience (Borghi et al. 2019; Mazzuca et al. 2025b). This does not mean that abstract concepts do not recruit sensorimotor and linguistic representational networks (Sakreida et al. 2013). In fact, both concept types might be better understood as points in a multidimensional space (Troche et al. 2014). One key difference, however, between abstract and concrete concepts remains the referent. A concrete concept such as “table” has a relatively stable perceptual referent that interlocutors can point to, see, or manipulate. By contrast, an abstract concept such as “freedom” does not have a single stable perceptual referent, making its meaning more variable, perspective-dependent, and open to negotiation, as it is shaped by social and cultural contexts.

Because the meanings of abstract concepts tend to be more variable and contestable, discussing them may require closer monitoring of the interlocutor’s representations and epistemic state (Gandolfi et al. 2023). Abstract concepts are rated higher with respect to the importance of social metacognition (Villani et al. 2019), defined as the extent to which individuals rely on others to understand word meaning. Participants ask for more hints when guessing abstract concepts and show greater synchrony with the avatar providing these suggestions compared to the one assisting with concrete concepts (Fini et al. 2021). This social reliance in the context of acquiring abstract concepts is not limited to early development when children monitor others as sources of information when reasoning about concepts such as God, death, or life (Harris and Koenig 2006). Adults likewise depend on others to negotiate word meanings (Mazzuca and Santarelli 2022).

By conceptualizing dialogue as a joint activity conducted within a shared workspace of contextual information (Pickering and Garrod 2021a, b), and in the context in which abstract concepts provide fewer stable external referents, one can understand how achieving conversational alignment may require interlocutors to monitor one another’s interpretations and epistemic states more closely in the case of abstract concept conversations. Although the processing of abstract concepts is hypothesized to be particularly sensitive to other people’s knowledge and interpretations, much more than that of concrete concepts, and evidence largely supports such an interpretation, the relationship with specific processes of social cognition remains underexplored. One reason for this is the difficulty inherent in studying concepts in contexts similar to those of everyday interactions.

One notable exception is the conversational paradigm developed by Villani et al. (2022), in which participants produce text-based responses to concept-matched prompts from virtual interlocutors, with abstract and concrete concept subtypes originally validated by Villani et al. (2019). This paradigm served as an inspiration for the present endeavor: its core conversational dynamics have been successfully replicated and extended beyond the original sample in a preregistered behavioral study demonstrating that abstractness systematically shapes conversational alignment and social monitoring (Mazzuca et al. 2025a). However, we employed the interactive conversational paradigm developed by Villani et al. (2022) as the encoding phase of a modified source memory task (Davachi 2006; Johnson et al. 1993; Yonelinas 2001, 2002) in order to investigate the way in which memory of social stimuli is impacted by grounding abstract concepts in conversation. This is a first step towards disentangling the mechanisms of acquisition and representation of abstract concepts, which requires a brief excursus on the recognition memory associated with social stimuli.

## Social recognition cues

According to the WAT framework, abstract concepts activate a social search process: because their meanings are less determinate and more contestable than those of concrete concepts, speakers are motivated to monitor and utilize interlocutors as epistemic sources (Borghi et al. 2019; Shea 2018; Gandolfi et al. 2023). Critically, this heightened social monitoring may recruit deeper, more elaborative processing of the interlocutor’s identity, in line with Craik and Lockhart’s (1972) levels-of-processing framework, whereby encoding that involves greater semantic elaboration produces stronger and more durable memory traces. If abstract concepts recruit more social-cognitive resources during interaction, such engagement may have downstream consequences for how social information is encoded and retained.

Davis et al. (2022) provide relevant evidence for the role of contextual information in abstract concept processing, while also showing that not all contextual cues are encoded equally. Using source-memory and context-reinstatement tasks, they provide evidence that arbitrary episodic contexts, such as speaker voice or coloured frames, are remembered less well for abstract than for concrete concepts, and do not confer a recognition benefit for abstract words when reinstated at test. By contrast, when the context was spatial location, retaining the same context produced a recognition-memory advantage for abstract concepts. These findings are informative to the extent that, besides the salience of spatial location, there is initial evidence for a slight advantage of voice over colors, which the authors explain through the fact that voice can act as an identity cue deemed to be more informative and, thus, less inhibited in the process of abstract conversation context retention. The present study further explores this logic connected to social identity cues, asking whether faces and names become more strongly encoded when interlocutors are encountered during abstract concept discussions.

Faces and names were chosen to investigate this selective attunement of social stimuli through their discreteness, which allows for manipulation, and through their pervasiveness. Faces occupy a privileged position among social cues: they are processed rapidly and evaluated automatically for trustworthiness, competence, and emotional state, even at minimal exposure (Todorov et al. 2009; Todorov 2012; Mason et al. 2006). Crucially, these evaluations are not passive: they are socially motivated, driven by the perceiver’s need to assess whether the interlocutor is a reliable and relevant epistemic source (Ghuman et al. 2014). In a conversation about abstract concepts, where the interlocutor’s knowledge, values, and perspective bear directly on the meaning being negotiated, this epistemic orientation should be heightened: attention to the interlocutor’s face (as the primary signal of their identity and credibility) may be greater and more sustained, resulting in better subsequent recognition. Names also carry identity information, even though they might be deemed more arbitrary social labels that do not convey the same density of diagnostic social signals. In the context of abstract concept dialogues, they can act either as anchors or as empty containers which are useful through their associations with other possible social stimuli encountered while monitoring others.

This rationale motivates examining whether concept type differentially affects the encoding and subsequent recognition of social stimuli, with a potentially stronger effect for faces than for names because faces convey richer socially relevant information. In this regard, while faces have traditionally been considered easier to remember and more salient than names (Young et al. 1985), more recent evidence suggests that methodological differences across studies largely drive this apparent advantage: faces are typically tested via recognition tasks, whereas names are more often assessed through recall (Burton et al. 2019). In light of these findings, we chose to assess recognition memory for both types of social stimuli. This does not mean that one cannot expect differences between the two types of stimuli, particularly as the experimental paradigm we propose does not focus on concepts in isolation as typical memory and linguistic studies do, but aims to understand social cognitive mechanisms in a situated, interactive context.

The cooperative, meaning-making nature of abstract-concept conversations may also influence the perceived quality of the relationship. According to Uncertainty Reduction Theory (Berger and Calabrese 1975), individuals who engage in active, informationally rich exchanges tend to experience greater interpersonal closeness with their interlocutors. Discussing abstract concepts, which are more contested, open-ended, and reliant on the interlocutor’s input for grounding, may therefore generate a stronger sense of epistemic partnership and perceived psychological closeness than discussing concrete concepts, whose meanings are generally more strongly constrained by perceptual and action-related information and require less collaborative elaboration.

## The present study

In the present study, we implemented an experimental paradigm consisting of an encoding and a recognition task within a computer-mediated communication (CMC) context of written, asynchronous textual exchanges (Walther 1996; Tidwell and Walther 2002). In such a context, visual social cues are largely reduced to static profile pictures and usernames (Sproull and Kiesler 1986; Spears and Lea 1992), making these minimal cues disproportionately salient in identity formation and social impression (Tanis and Postmes 2003; Bacev-Giles and Haji 2017; Walther et al. 2008), which further renders names and faces the natural focus of a social memory paradigm in our setting. For the present study, this CMC context was particularly suitable because, on the one hand, it reduces the number of cues available in the shared workspace by rendering nonverbal and paraverbal signals largely unavailable (Sproull and Kiesler 1986; Shockley et al. 2009), and on the other hand, it increases the prevalence of more direct and personal uncertainty-reduction behaviors (Tidwell and Walther 2002).

Our experiment builds on the conversational paradigm developed by Villani et al. (2022) in order to preliminarily assess whether concept types and subtypes exhibit the same psycholinguistic associations in Romanian as those previously validated in Italian (Villani et al. 2019) and adopted in the present study. In Romania, feature elicitation and behavioral studies validating specific abstract–concrete distinctions are largely absent. We validated the relevant dimensions, in this case, the abstract–concrete scale starting from Villani et al. (2019), who identified subtypes of Italian abstract concepts including physical, spatio-temporal, and quantitative concepts, philosophical or spiritual concepts, and notions related to the self, social interaction, emotional and inner-state concepts, and subtypes of concrete concepts including tools, animals, and food. As each subtype is associated with partially distinct neural activation patterns (Wilson-Mendenhall et al. 2013; Mellem et al. 2016), it is theoretically relevant to compare the conversational dynamics elicited by different concept types and subtypes, as well as their impact on conversational strategies and linguistic features. Although the present validation is preliminary, effect-level and, therefore, context-sensitive, it nonetheless provides evidence beyond the assumption that abstractness and concreteness map identically across languages. We treat it as an embedded manipulation check within the encoding phase of the social memory task, a prerequisite for the validity of any social psycholinguistic investigation, which may also be useful for researchers studying other languages in which psycholinguistic resources remain underdeveloped.

In the simulated exchanges, each prompt was presented as a message authored by a particular virtual agent, the person with whom the participant was ostensibly engaging, whose face and name identified the interlocutor. After interacting with agents in a simulated online environment, participants were asked to recognize the names and profile pictures of the interlocutors, as if the interaction had taken place in a real-world context. Participants were also asked whether they would be willing to continue the conversation with the recognized interlocutors to assess whether the type of concept discussed modulated the interpersonal dynamics generated during the interaction. Previous work reported a numerical trend suggesting differences in perceived psychological or physical closeness as a function of concept type, although this effect was not subsequently replicated (Fini et al. 2023).

To conclude, our paradigm comprises two functionally integrated components. In the first step, we aim to replicate the findings of Villani et al. (2022) in order to preliminarily validate the selection of sentences containing different types of concepts. In the second, and more important, step, we focus on the differential social episodic memory and perceived psychological closeness modulated by the processing and conversational representation of ACs and CCs. These two components motivate the following hypotheses:

### For conversational dynamics (manipulation check)

H1: Participants will use more uncertainty expressions or signs of uncertainty (uncertainty expressions, questions, repetitions of target word) in the abstract concept (AC) condition than in the concrete concept (CC) condition.

H2: Participants will display greater interpersonal reliance in the AC condition than in the CC condition, which manifests in continuing the discussion by assigning a further turn to the other.

H3: Participants will use more general statements in the AC condition than in the CC condition.

H4: Participants will use more internal situational elements such as producing emotional, introspective, belief, or metacognitive properties when discussing ACs as opposed to CCs.

### For social memory and closeness

H5: The names of avatars from the AC condition will be recognized more than the names of avatars from the CC condition.

H6: The profile pictures of avatars from the AC condition will be recognized more than the profile pictures of avatars from the CC condition.

H7: Avatars from the AC condition will be preferred as opposed to avatars from the CC condition for further face-to-face conversations.

## Methodology

### Materials

#### Names

A total of 70 first name and last name combinations of similar lengths (M = 12.27, SD = 2.39, MIN = 7, MAX = 20) were created by consulting the available lists with the most common Romanian names for either of these two categories (Ravara 2024; Name Statistics Romania, n.d.). We selected 35 female names and 35 male names. They represented the most frequent names from each category (e.g., *Ana Ionescu* - female name, *Mihai Popescu* – male name). First and last names were associated using a simple randomization process. The same process was used to allocate 60 of these name combinations for the encoding phase and 10 for the recognition phase. The latter were used as decoys along with 10 other name combinations that were randomly selected from the ones presented earlier to the participants in the first phase.

#### Faces

A total of 70 faces were selected from the Chicago Face Database’s main set (Ma et al. 2015). The CFD dataset includes 597 high-resolution, standardized photographs of males and females of various ethnicities having different facial expressions. We selected only the White female and male models, in equal proportions, because of the Romanian context, in which there are fewer Asian, Black, and Latino individuals, and because we wanted to offer a somewhat more realistic match within the Romanian sociocultural context. We wanted to simulate a social media environment in which profile pictures are typically neither neutral nor negative from an emotional perspective, which is why we have selected the photos in which the persons have a happy, covert, mouth-closed expression. Simple randomization was used to select exactly 70 pictures that met all these conditions. The minimum rated age was 16.96, and the maximum rated age was 37.59 (M = 26.24, SD = 4.62). Similar to the procedure for selecting the name combinations, 60 photographs were used for the encoding phase and 10 for the recognition phase as decoys. Out of the 60 photographs used in the first phase, 10 were chosen using simple randomization to appear in the next phase as well.

#### Conversational prompts

The conversational prompts were adapted from Villani et al. (2022) in Romanian by bilingual speakers using the English translations provided by Villani et al. (2022) (see Table 1 for a sample of conversational prompts). These consist of 60 sentences composed of a verb and a noun denoting either an abstract or a concrete concept, which have similar word lengths (M = 19.78, SD = 2.48). Each type is equally distributed: 30 abstract concepts and 30 concrete concepts. In turn, each of these includes 3 sub-categories: for the concrete concepts there are 10 tool concepts (e.g. hammer, umbrella, bottle, pencil, etc.), 10 animal concepts (e.g. dog, lion, sheep, camel, etc.), and 10 food concepts (e.g. banana, tomato, cake, grapes, etc.), while for abstract concepts there are 10 philosophical-spiritual concepts (PS, e.g. moral, destiny, salvation, paradise), 10 physical, spatial-temporal and quantitative concepts (PSTQ, e.g. number, acceleration, result, schema, etc.), and 10 emotional-social concepts (EMSS, e.g. shame, joy, friendship, revenge, etc.). The sentences that involve declarative statements in the first person, adapted from Italian into Romanian, have been preliminarily validated in the first step of our study (see Results and Discussion).

**Table 1:** Sample conversational prompts translated from Villani et al. (2022)

Type of concept	Message in Romanian	Message in English
Concrete (Animals)	Am fotografiat leul.	I photographed the lion.
Concrete (Animals)	Am auzit papagalul.	I heard the parrot.
Concrete (Foods)	Am achiziționat strugurii.	I bought the grapes.
Concrete (Foods)	Am condimentat roșia.	I seasoned the tomato.
Concrete (Tools)	Am aprins lampa.	I turned on a lamp.
Concrete (Tools)	Am folosit bormașina.	I used the drill.
Abstract (Physical - Spatial - Temporal - Quantitative)	Am calculat numărul.	I calculated the number.
Abstract (Physical - Spatial - Temporal - Quantitative)	M-am gândit la bani.	I thought about money.
Abstract (Philosophical - Spiritual)	Am visat paradisul.	I dreamed of paradise.
Abstract (Philosophical - Spiritual)	Am rezolvat o enigmă.	I solved an enigma.
Abstract (Emotions, Mental States and Sociality)	Mi-a fost rușine.	I felt ashamed.
Abstract (Emotions, Mental States and Sociality)	Am evitat un conflict.	I avoided a conflict.

### Participants

For participant selection, we used a screening questionnaire in Google Forms to check beforehand that participants met all conditions for the proper functioning of the experiment. These included having access to a laptop or a computer and a good internet connection because the experiment was to be taken online under these conditions. Along with these, demographic questions and a couple of questions related to their social media usage were introduced. Participants who met all the conditions were subsequently sent an email with the login details and the link for the experiment. The form included 73 participants, out of which one was excluded since they did not own a computer. This was necessary because the experiment was not designed for other devices. Only 40 participants completed the experiment.

The final sample comprised 40 native Romanian speakers aged 19–49 years (M = 25.19, SD = 8.75), including 25 women, eight men, and three non-binary participants. The highest education level for most participants was high school (*n* = 26), with fewer participants who had graduated from university (*n* = 6) or had finished a postgraduate degree (*n* = 4). 4 participants did not follow the instructions to write the identification code they were allocated, which was intended to help the researchers connect the two data sources. They could not be contacted afterward to repair the issue, which is why demographic data is missing for these participants.

We used Bayesian Generalized Linear Mixed Models for both the replication (the first component of the study) and the extension. Bayesian analyses do not typically require a sample size decided beforehand for hypothesis testing, as frequentist tests do. Nevertheless, we consulted previous literature and found studies that investigated differential behavioral effects between abstract and concrete concepts, employing from 21 to 80 participants (Zdrazilova et al. 2018; Fini et al. 2021; Villani et al. 2022). We had a retention rate of 54.8% from the 73 participants who passed the screening, but the final sample size (*n* = 40) remained within the interval we needed to investigate the desired effects.

All participants were naive to the whole purpose of the experiment, took part voluntarily, and received no compensation for their participation. They were informed only that it aimed to study the dynamics of online conversations, as knowledge of the memory task might have influenced their top-down attentional processes and, subsequently, the results. The study was conducted in accordance with the Declaration of Helsinki (World Medical Association 2013). All participants provided informed consent prior to participation and were free to withdraw at any time.

### Procedure

**Fig. 1:**
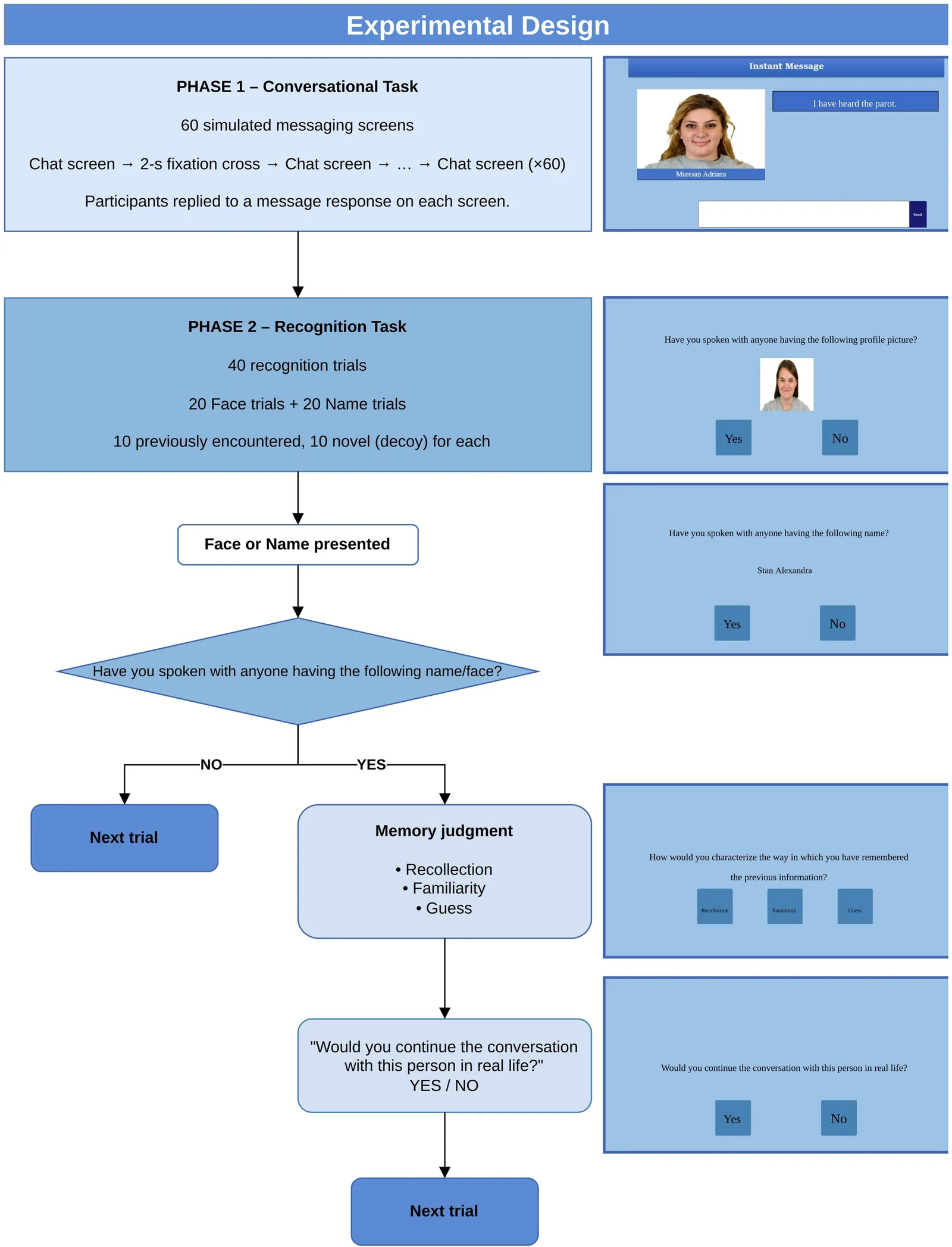
The figure illustrates the sequence of the conversational and recognition tasks. The text displayed on the experimental screens has been translated into English; during the experiment, all instructions, prompts, and response options were presented in Romanian. Examples of the original Romanian conversational prompts and their English translations are provided in Table 1

We developed the experiment with the help of PsychoPy3 (Peirce et al. 2019) and launched it online via Pavlovia. The experiment consisted of two parts and employed a within-participants design, with concept type (abstract vs. concrete) as the primary within-subjects independent variable. In the first phase, participants engaged in a conversational task that served as the encoding phase for the stimuli presented on screen. They were instructed to imagine that they were using an online messaging platform and had to reply to messages from multiple users as if they were familiar with them and as if the conversation took place in an everyday context. During the conversational task, all participants responded to 60 message prompts (i.e., 30 abstract concepts and 30 concrete) presented in random order within an interface simulating an online messaging application (see Fig. 1). Each prompt was accompanied by the first and last name of an interlocutor, along with a clearly visible profile picture. The pairing of prompts, names, and images was fully randomized. The second phase was a recognition task, which also included questions assessing participants’ willingness to continue a conversation with the interlocutors they recognized. At the end, participants were debriefed, and their understanding of the recognition task was verified.

The study was not preregistered; however, the materials, datasets, and analysis scripts are openly available on OSF (https://osf.io/9hqcy/overview?view_only=3b3852b4d9804533a3ada3ad92e4a535).

Participants were asked to reply to the given messages in writing, simulating an online conversation that could happen between them and someone they know in a real-life setting. They were provided with examples illustrating possible responses in either affirmative, negative, or interrogative sentences. No character limit was imposed, but a time limit was set at 30 s per prompt. This time constraint was intended both to control for encoding effects, since longer exposure could enhance recognition, and to ensure that the entire experiment duration remained under one hour, minimizing participant fatigue and facilitating recruitment and engagement.

The recognition task took place in the second phase of the experiment. Participants were randomly presented with 20 stimuli from each category - names and faces - including 10 targets and 10 decoys per category. The decoys consisted of name–surname combinations and profile pictures that had not appeared during the encoding phase. Participants were asked whether they had previously interacted with the person whose name or face appeared on screen. They could answer either by pressing a “Yes” or a “No” button. Afterward, if the participants recognized the interlocutor, they were asked whether their recognition was based on remembering, knowing, or guessing (Gardiner et al. 1999).

To prepare participants for the remember/know/guess classification, they were instructed as follows: a “remember” response was appropriate when recognition was based on a specific recollection from the encoding phase - such as recalling the associated face or conversation; a “know” response indicated confidence in recognition without awareness of contextual details; and a “guess” response was to be used only when neither applied. The inclusion of the “guess” option was based on evidence that it improves the validity of “know” responses (Gardiner et al. 1999). At the end of the task, participants’ understanding of these categories was assessed through three multiple-choice questions. All participants correctly answered all three and were included in the final sample. In the subsequent analyses, ‘know’ responses are referred to as familiarity-based recognition, and ‘remember’ responses as recollection-based recognition, following standard dual-process terminology (Yonelinas 2001, 2002).

Each sequence of these two recognition questions was followed, if the answers were positive, by a question about whether the participants would want to continue the conversation with their interlocutor in a real-life, in-person setting. Participants could reply by pressing a “Yes” or “No” button. This binary item was treated as a task-specific proxy for willingness to continue the interaction and, more cautiously, perceived interpersonal closeness. As already mentioned, we expect different results here based on the type of concept discussed. Previously, Fini et al. (2023) found a numerical trend that psychological or physical closeness changed as a function of the type of concept discussed, but it was not subsequently replicated.

### Sentence coding

Nine features have been chosen for the preliminary, context-sensitive validation of the abstract and concrete words we used in the current study, all corresponding to specific conversational dynamics that each concept type or subtype elicits and that we consider relevant to the main hypotheses (see Table 2 for the selected features). The features have been taken from Villani et al. (2022), which in turn were adapted from Barsalou and Wiemer-Hastings (2005) and Zdrazilova et al. (2018). Two professional coders were involved, one of whom was blind to the study’s aims. The computed reliability between them was 96.83%. The disagreements were resolved with the help of a third judge. The number of words and the occurrence of the target concept word in the body of the messages have been computed automatically.

**Table 2:** Conversational and linguistic features analyzed

Feature name	Definition	Example
Number of Questions	The number of questions produced by participants for the target sentence.	-
Uncertain expressions	Participants express doubt, uncertainty, and requests for clarification.	e.g. mmh?, and so?, what do you mean?
General statement	Participant is expressing a statement, a general opinion, or common knowledge.	e.g. I felt the calm: It’s a beautiful feeling; I understood the reason: It’s fundamental; I did a kindness: Empathy is a human feeling.
Turn-taking	Participant is trying to establish a conversation, for example asking questions or using expressions signaling the willingness to know more details. Turn includes expressions; no turn was coded when participants expressed a simple, short reply or a general statement.	e.g. and?, Tell me, I listen to you, Do you want to talk about it?
Repetitions of target word	The number of repetitions of target words produced for each sentence.	e.g. I saw a cow – Where did you see the cow?
Emotion	Participant refers to an emotional/ affective state.	e.g. I am not vengeful, I am happy, I was ashamed, How do you feel?, I am glad, I hope it works.
Metacognition	Participant refers to their own cognitive processes and mental operations.	e.g. I estimated the acceleration: I am not able to study physics/ I am good at memorising; I had a sympathy: I remember when I thought of him.
Belief/intentions	Participant is expressing a belief explicitly or implicitly.	e.g. I think that…, In my opinion…, I believe, I need it, I should do that, You don’t do that, It was necessary.
Introspection	Participant examines their memories, feelings, and experiential states that the speaker is aware of.	e.g. I made a cake: I remember when my grandmother prepared my birthday cake; I felt ashamed: I often feel ashamed when I speak in public; I overcame the fear: I am sure my fear of insects is exaggerated, but I can’t control it.

### Debriefing responses

At the end of the experiment, participants were encouraged to give feedback related to their experience in an open-ended question format. They were given anchors on what aspects they could focus on in their responses: what they thought about or felt while they were participating in the experiment or afterward, what they think the research is about, or any other input related to their experience. Interestingly and in a sense contrary to the results, 30% of participants reported that they were not attentive to either the faces or the names because their main focus was to write the messages. 12.5% of the participants found the messages to be slightly unrealistic in their simplicity, formality, or lack of context. In comparison, 10% of the participants found the profile pictures to be unrealistic or strange. 12.5% of participants explicitly reported that they liked the experiment and felt engaged. None of the participants correctly guessed the aims of the study. The percentages presented here are provisional precisely because they are calculated based on the information participants decided to share.

### Analysis

We have run Bayesian generalized linear mixed models for the hypotheses related to the preliminary validation and the recognition memory task. This is because we aim to propose an experimental paradigm that is easy to reproduce and to build upon in the context of the relationship between social interaction, social cognition, and specific types of concepts. For this reason, we have not focused on large sample sizes and instead chose to provide initial evidence for the hypothesized effects and the utility of the experimental procedure. Bayesian analyses were fitting for these reasons because they are more flexible than frequentist counterparts, allow for cumulative research, do not require large sample sizes, and make it possible to reach graded conclusions needed for such preliminary work.

Bayesian Generalized linear mixed models estimate the probability of a given model’s parameters taking different values, given the observed data. Bayes’ theorem captures the relationship between prior expectations and the likelihood of them changing if different parameter values were chosen. The posterior density function is generated from the prior and the likelihood. A new sample is generated and used to establish the 95% credible intervals, which are the range of values that have a 95% probability of containing the true value for a given parameter. When such an interval does not contain zero, we treat it as credible and worth interpreting.

For preliminary validation, we aimed to replicate the conversational effects of Villani et al. (2022), so we ran the same types of analyses. For each hypothesis, we computed two models based on the two different predictors: Type of Concept (Abstract vs. Concrete) and Subtype of Concept (concrete: Tool, Food, Animals; abstract: Philosophical-Spiritual/PS, Physical-Spatio-Temporal-Quantitative/PSTQ, Emotional-Mental State-Social/EMSS). It is appropriate to include both in the same model, as these within-subject factors are strongly correlated. We have chosen only one of the two models for the inferential statistics by comparing the best fit as shown by the Bayes factor. The Bayes factor is the ratio of the probabilities of the data, which shows how much prior odds change, given the data (Kruschke 2015). We have considered, by convention, that there is “substantial” evidence for model 1 when the BF exceeded 3.0 and for model 2 when the BF was less than ⅓ (Kruschke 2015).

A Poisson distribution with a logistic link function was used for the number of questions and repetition of target words. In contrast, a Binomial distribution with a logit link function was used for all others from the conversational task. All models except the Null Hypothesis ones were run in the same way as in Villani et al. (2022) by using a custom set of computing four sampling chains, each with 10,000 iterations: the first 4000 of these being treated as warm-ups, resulting in 24,000 posterior samples. The *adapt_delta* and *max_treedepth* parameters were set to 0.99 and 15 to increase the sampler’s performance. The Null Hypothesis or intercept-only models were run using a set of four sampling chains, each with 5000 iterations, with the first 2000 being warm-ups. These have resulted in 12,000 posterior samples. The *adapt_delta* and *max_treedepth* parameters were set in the same way as in Type and Subtype of Concept models.

All models were fit using the intercept priors from Villani et al. (2022). These are the following: β_uncertainty_∼ N(− 2.68,17.33), β_questions_∼ N(− 0.57,0.06), β_target_words_∼ N(− 2.73,0.19), β_turn−taking_∼ N(0.34,0.1), β_general_statements_∼N(− 3.41,0.21), β_emotion_∼ N(− 3.2,0.19), β_introspection_∼ N(− 4.44,0.4), β_belief_∼ N(− 3.1,0.18), β_metacognition_∼ N(− 49.87,84.28). For the slope and other parameters, we used non-informative priors to allow the data to inform these aspects of the model freely, precisely because we aimed to compare the conversational dynamics of Italian and Romanian language speakers as generated by different types and subtypes of concepts. All models were reliable, reaching convergence with a potential scale reduction factor on split chains (R.hat) of 1.00 and suitable effective sample size measures, as evaluated with the *Bulk_ESS* and *Tail_ESS* components. The hypothesized differences between AC and CC conditions were investigated with a contrast method.

Each Bayesian Generalized Linear Mixed Model included random subject intercepts to account for subject-level variation (Zdrazilova et al. 2018). For the recognition memory task, we employed four Markov Chain Monte Carlo chains with 2,000 iterations each (1000 warm-up, 1000 post-warm-up). Convergence was reached quickly, as confirmed by R.hat values close to 1.00 and adequate effective sample sizes (*Bulk_ESS* and *Tail_ESS*). For the binary outcomes, such as name and face recognition and perceived closeness, we used a Bernoulli likelihood with a logit link. In contrast, for the categorical outcomes, namely the recognition type for names and faces, respectively, we used a categorical likelihood with separate logit links for each non-baseline response category. The decoy condition served as the baseline. We used default priors for these analyses, as we did not find any strong prior information available; these are weakly informative and thus flexible.

Analyses were carried out in R (version 4.4.0; R-Core Team, 2024) using packages such as “openxlsx” (Schauberger and Walker 2020), “dplyr” (Wickham et al. 2021), “brms” (Bürkner 2017) for fitting Bayesian mixed-effects models using the Stan programming language, and “aov” (Chambers et al. 1992) for fitting the analysis of variance models. The bar charts on polar axis graphs were created using Matplotlib and NumPy libraries in Python (version 3.10.12). The dataset and the analysis code are available on OSF (https://osf.io/9hqcy/overview?view_only=3b3852b4d9804533a3ada3ad92e4a535) for further analyses, including frequentist estimations for meta-analysis inclusion.

## Results

### Preliminary validation of abstractness and concreteness in conversational dynamics

#### Hypothesis 1

**Fig. 2:**
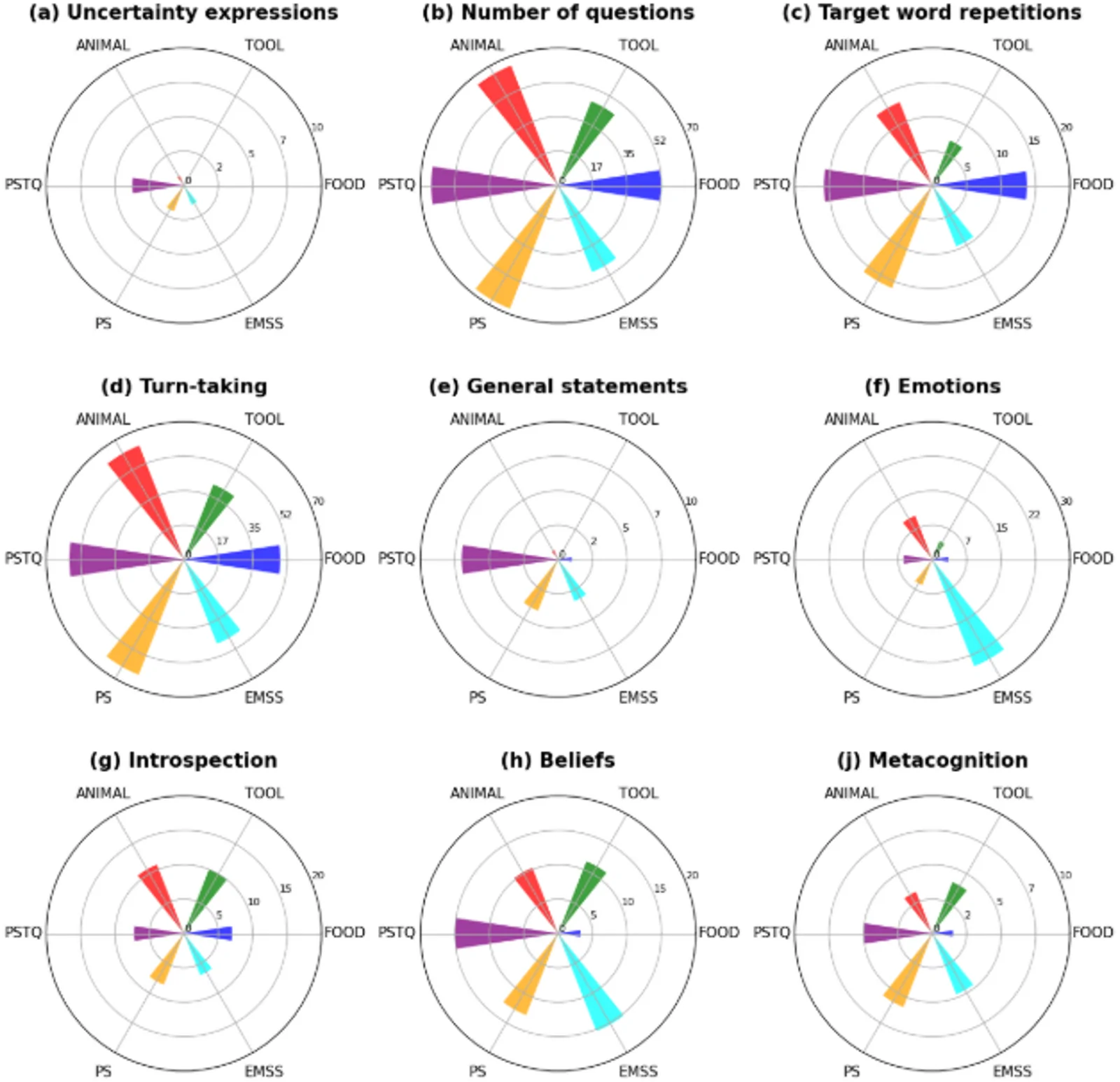
Frequency of socio-linguistic features within subtypes of concept. In this plot, *PSTQ* Physical-spatio-temporal quantitative concepts, *PS* Philosophical-spiritual concepts, *EMSS* Emotional mental states and social concepts

According to our first hypothesis, participants will use more uncertainty expressions or signs of uncertainty (uncertainty expressions, questions, repetitions of the target word) in the abstract concept (AC) condition than in the concrete concept (CC) condition. The frequency of socio-linguistic features across concept subtypes is displayed in Fig. 2.

**Uncertainty expressions.** By comparing the Bayes Factor (BF) of the three models run (Type of Concept, Subtype of Concept, Null Hypothesis Model), we detected that the Subtype Model had the best fit. Upon further analysis of conditional effects, the mean probability of sentences with food, animals, EMSS, PSTQ, PS, and tool concepts had an Uncertainty Expression of 0.53%, 0.61%, 1.14%, 2.96%, 1.53%, and 0.21%, respectively. The difference between ACs and CCs in terms of being encoded with more Uncertainty Expressions was not strong: the median estimate was approximately 1.1%, 95% CI [0.5,1.8]. By inspecting the mean number of posterior observations major to zero, we were up to 100% confident that Uncertainty Expressions are more frequent in ACs than CCs. The contrast method was used to investigate whether Uncertainty Expressions were more frequent in PSTQ concepts than in EMSS and PS concepts. The more credible difference that PSTQ was encoded with more Uncertainty Expressions than other abstract concepts was 1.56%, 95% CI, [0.10, 3.53]. The mean number of posterior observations major to zero, however, pointed to a 98.21 confidence that Uncertainty Expressions in PSTQ concepts were more frequent than in EMSS and PS concepts.

**Number of questions.** The Concept Subtype Model was the one that fit best for the number of questions. In a further analysis of conditional effects, the mean probabilities of sentences with food, animal, EMSS, PSTQ, PS, and tool concepts by number of questions were 51.21%, 64.83%, 47.00%, 62.88%, 65.30%, and 45.53%, respectively. The difference between ACs and CCs in terms of being encoded with a higher number of questions was moderate/strong (4.39%, 95% CI [-0.22, 9.08]). By inspecting the mean number of posterior observations greater than zero, we were up to 96.88% confident that there were more questions used by participants when discussing ACs as opposed to CCs. The contrast method was used to investigate whether questions were more frequent in animal concepts than in the other concrete concepts, on the one hand, and whether they were more present in the PS concepts as opposed to the other abstract concepts. For the first hypothesis, animal concepts were encoded with more questions than tool or food concepts, with a credible difference of 4.39%, 95% CI [-0.2,9.08]. The mean number of posterior observations major to zero pointed to a 96.87% confidence that there were more questions in animal than in food and tool concepts. For the second hypothesis, the credible difference between PS and EMSS and PSTQ concepts was 10.5%, 95% CI [0.22, 19.8]. We have computed the mean number of posterior observations major to zero which pointed to a 98.64% confidence that more questions were used for PS as opposed to other ACs.

**Repetitions of target words.** Through comparison, we found that the model with the Subtype of Concept is the one that fit best. Further analysis of conditional effects showed that the mean probability of sentences with food, animal, EMSS, PS, PSTQ, and tool concepts had repetitions of target words of approximately 8.5%, 8.14%, 5.97%, 9.84%, 10%, and 4.43%, respectively. The difference between ACs and CCs in terms of being encoded by making use of more target word repetitions was not strong (0.94%, 95% CI [-0.39, 2.4]). By inspecting the mean number of posterior observations major to zero, we were up to 91.92% confident that repetitions of target words were more frequent in ACs than CCs. The contrast method was used to investigate whether repetitions of target words were more frequent in PSTQ than in PS or EMSS concepts. The more credible difference that PSTQ concepts were encoded with more target word repetitions than other abstract concepts was 1.80%, 95% CI, [-0.97, 4.95]. The mean number of posterior observations major to zero, however, pointed to an 89% confidence that repetitions of target words in PSTQ concepts were more frequent than in EMSS and PS concepts.

#### Hypothesis 2

The second hypothesis states that participants will display greater interpersonal reliance in the AC condition than in the CC, which manifests in continuing the discussion by assigning a further turn to the other.

**Turn-taking.** The Subtype of Concept is the one that fit best after comparing the Bayes Factors of the three models. The mean probability of sentences with food, animal, EMSS, PSTQ, PS, and tool concepts to have turn-taking was 51.96%, 66.22%, 47.56%, 62.58%, 66.49%, and 43.19%, respectively. The difference between ACs and CCs in terms of being encoded by assigning a turn to the interlocutor was moderate (4.9%, 95% CI [1.67, 8.30]). By inspecting the mean number of posterior observations major to zero, we were up to 99.83% confident that assigning a further turn in the conversations is more frequent in ACs than CCs. The contrast method was used to investigate whether turn-taking is more frequent in PS than in PSTQ or EMSS concepts. The more credible difference that PS concepts are encoded with more turns assigned to the interlocutor than other abstract concepts is 5.55%, 95% CI [0.43, 11.54]. The mean number of posterior observations major to zero pointed to a 96% confidence that turn-taking happened more while discussing PS concepts as opposed to EMSS or PSTQ concepts.

#### Hypothesis 3

Next, we investigated whether participants would use more general statements in the AC condition than in the CC condition.

**General statements.** The Subtype of Concept was chosen between the three models because it had the best performance. In the analysis of conditional effects, the mean probabilities of sentences with food, animal, EMSS, PSTQ, PS, and tool concepts having general statements were approximately 1.66%, 1.05%, 4.13%, 6.37%, 4.12%, and 0.88%, respectively. The difference between ACs and CCs in terms of being encoded with general statements was small (2.61%, 95% CI [1.56, 3.92]). By inspecting the mean number of posterior observations major to zero, we were up to 100% confident that using general statements was more frequent in ACs than CCs. The contrast method was used to investigate whether general statements are more frequent in PSTQ than in PS or EMSS concepts. The more credible difference that PSTQ concepts were encoded with more general statements than other abstract concepts is 2.18%, 95% CI [0.26, 5.10]. The mean number of posterior observations major to zero indicated 95.9% confidence that general statements were used more while discussing PSTQ concepts than EMSS or PS concepts.

#### Hypothesis 4

For the fourth hypothesis, we explored whether participants would use more internal situational elements, such as producing emotional, introspective, belief, or metacognitive properties, when discussing ACs as opposed to CCs.

**Emotions.** The best fitting model was the Concept Subtype. The mean probabilities of sentences with food, animal, EMSS, PSTQ, PS, and tool concepts to denote emotions were approximately 2.84%, 6.79%, 21.74%, 4.42%, 3.20%, and 3.04%, respectively. The difference between ACs and CCs in terms of being encoded with emotions was strong (6.73%, 95% CI [4.90, 8.79]). By inspecting the mean number of posterior observations major to zero, we were up to 100% confident that emotions were more frequent in ACs than CCs. The contrast method was used to investigate whether emotions were more frequent in EMSS than in PS or PSTQ concepts. The more credible difference that EMSS concepts were encoded with more emotions than other abstract concepts was 17.8%, 95% CI [13.12, 23.25]. The mean number of posterior observations major to zero indicated 100% confidence that emotions were more frequent while discussing EMSS concepts than PS or PSTQ concepts.

**Introspection.** By comparing the Bayes Factor of the three models run, we identified that neither provided substantial evidence. Nevertheless, we chose to report the model with the Type of Concept because this fit best. In an analysis of conditional effects, the mean probability of sentences with abstract and concrete concepts having introspective properties was approximately 2.81% and 2.36%. The difference between ACs and CCs in terms of being encoded with introspective properties was inconclusive (-0.4%, 95%CI [-1.44, 4.31]). By inspecting the mean number of posterior observations major to zero, we are up to 15% confident that using introspective properties is more frequent in ACs than CCs.

**Belief.** The Subtype of Concept was the best-fitting model. The conditional effects analysis returned the mean probabilities of sentences with food, animal, EMSS, PSTQ, PS, and tool concepts to be encoded with beliefs: 2.75%, 6.05%, 10.57%, 9.82%, 8.17%, and 7.44%, respectively. The difference between ACs and CCs in terms of the presence of overt beliefs was small (3.33%, 95% CI [1.83, 5.14]). By inspecting the mean number of posterior observations major to zero, we were up to 100% confident that beliefs were more frequent in ACs than CCs. One contrast method was used to investigate whether beliefs were more frequent in EMSS than in PS or PSTQ concepts. The more credible difference that EMSS concepts were encoded with more beliefs than other abstract concepts was 1.53%, 95% CI [-1.40, 4.87]. The mean number of posterior observations major to zero pointed to an 84% confidence that beliefs were made more overt while discussing EMSS concepts as opposed to PSTQ or PS concepts. Another contrast method was used to investigate whether there was a difference between tools and other concrete concepts when it comes to beliefs. The credible difference has been recorded as 2.98%, 95% CI [0.70, 5.74]. As shown by the mean number of posterior observations major to zero, there was a 99.58% confidence that beliefs were more frequent in discussing tools as opposed to concepts which denote animals or food.

**Metacognition.** Neither of the models run provided substantial evidence. Nevertheless, we chose the Subtype of Concept model to analyze the conditional effects because this model had the best fit. The mean probabilities of sentences with food, animal, EMSS, PSTQ, PS, and tool concepts having metacognitive properties were approximately 1.26%, 1.76%, 2.47%, 2.32%, 3.22%, and 2.18%, respectively. The difference between ACs and CCs in terms of being encoded with metacognitive properties was inconclusive (0.42%, 95%CI [-0.21,1.24]). By inspecting the mean number of posterior observations major to zero, we were up to 90.35% confident that using metacognition is more frequent in ACs than CCs. One contrast method was used to investigate whether metacognitive properties were more frequent in PS than in PSTQ or EMSS concepts. The more credible difference that PS concepts were encoded using metacognition more than other abstract concepts was 0.74%, 95% CI [-0.69, 2.13]. The mean number of posterior observations major to zero pointed to an 84.99% confidence that metacognitive properties were used more while discussing PS concepts as opposed to EMSS or PSTQ concepts. In addition, another contrast method was used to investigate whether there was a difference between tools and other concrete concepts when it came to metacognition. The credible difference was 0.61%, 95% CI [-0.45,2.13]. As shown by the mean number of posterior observations major to zero, there was an 86.62% confidence that metacognitive properties were more frequent in discussing tools as opposed to concepts which denote animals or food. These results must be taken with precaution as the model was not found to be substantial.

### Social recognition memory task

#### Hypothesis 5

The first hypothesis for the second part of the study held that the names of avatars from AC conditions would be recognized more than the names of avatars from CC conditions. The Bayesian logistic mixed-effects model fitted to recognition responses with the three conditions as a fixed effect and random intercepts for participants showed that relative to the decoy condition, both the concrete (β = 0.49, 95% credible interval [0.05, 0.91]) and the abstract (β = 0.29, 95% CI [–0.18, 0.75], odds ratio = 1.34) were recognized more. In the latter case, the effect is uncertain as the credible interval included zero. The contrast method indicated no reliable difference in recognition memory between AC and CC conditions (β = − 0.20, 95% CI [–0.71, 0.31], odds ratio = 0.82), with the credible interval spanning both negative and positive values.

We ran another model for the recognition type and compared the difference in familiarity and recollection responses between AC and CC conditions. Familiarity was higher for AC than for CC conditions (Estimate = 1.40, Est. Error = 0.58, 95% CI [0.28, 2.59]), with the 95% credible interval excluding zero, supporting the fact that agents from the AC conditions were recognized more based on familiarity. The difference in recollection responses was positive but not credibly different from zero (Estimate = 0.67, Est. Error = 0.38, 95% CI [− 0.03, 1.44]), indicating no reliable difference for recollection rates.

#### Hypothesis 6

Next, we investigated whether the profile pictures of avatars from AC conditions would be recognized more than those from CC conditions. Recognition memory of faces was significantly higher for both the AC conditions with an increase in log-odds of 1.20 (SE = 0.19, 95% CI [0.82, 1.58]) and CC conditions with an increase in log-odds of 0.74 (SE = 0.19, 95% CI [0.38, 1.12]), as opposed to the decoy baseline. The contrast analysis revealed a significant positive difference in recognition of faces (Estimate = 0.46, SE = 0.21, 95% CI [0.04, 0.88]), indicating that agents from AC conditions were better recognized than those in CC conditions.

For the recognition type of faces, we ran a categorical model and used the decoy conditions as the baseline. Recollection was significantly lower in AC as opposed to CC conditions (estimate = -0.72, SE = 0.27, 95% credible interval [CI] = [-1.24, -0.21]), the posterior probability exceeding 95%, while the difference in familiarity was not credible (estimate = -0.58, SE = 0.35, 95% CI = [-1.27, 0.09]), suggesting insufficient evidence to support a difference.

#### Hypothesis 7

For the last hypothesis, we investigated whether avatars from the AC condition would be preferred over avatars from the CC condition for further face-to-face conversations. We ran two models, one for each type of stimulus.

**Names.** The Bayesian hierarchical model fitted to predict participants’ perceived closeness to the agents with whom they briefly communicated in the AC or CC conditions revealed that neither condition significantly changed perceived closeness compared to baseline. Specifically, the posterior estimate for CC condition relative to baseline was 0.03 (SE = 0.26; 95% credible interval [CI] = [–0.50, 0.53]), and for AC condition relative to baseline was 0.36 (SE = 0.27; 95% CI = [–0.17, 0.89]), with both intervals including zero. By comparing directly AC and CC conditions, we obtained a posterior estimate of 0.33 (SE = 0.30; 95% CI = [–0.25, 0.92]). Although the posterior means suggest a trend toward higher perceived closeness in the AC compared to the CC condition, this finding is uncertain as the 95% credible interval spans zero.

**Faces.** The model’s posterior estimates indicated that, compared to baseline, both AC and CC conditions were associated with higher perceived closeness. The posterior estimate for the CC condition was 0.91 on the log-odds scale (SE = 0.23; 95% credible interval [CI] = [0.45, 1.36]), and the posterior estimate for the AC condition was 0.62 (SE = 0.23; 95% CI = [0.18, 1.08]). For both contrasts, the 95% CIs excluded zero, providing robust evidence that perceived closeness was higher in each condition relative to the decoy baseline. When directly contrasting the AC and CC conditions, the posterior estimate was − 0.28 (SE = 0.24; 95% CI = [–0.76, 0.19]), and the 95% CI included zero, indicating insufficient evidence for a reliable difference between the two conditions.

### Supplementary analyses

We ran supplementary analyses to better contextualize our findings. Firstly, we tested whether faces or names were recognized more accurately overall, collapsing across concept type. A one-sided Bayesian paired-samples *t*-test (default Cauchy prior, *r* = 0.707) provided evidence for the directional hypothesis that faces (*M* = 0.371, *SD* = 0.182) were recognized more accurately than names (*M* = 0.219, *SD* = 0.185), *BF*₋₀ = 13,817.04.

Next, we evaluated whether the face-recognition advantage could reflect greater processing effort during abstract-concept trials. For this, we submitted RTs to a Bayesian repeated-measures ANOVA with Phase (encoding, recognition) and Condition (concrete, abstract) as within-subject factors (default fixed-effects prior, *r* = 0.5). The model containing only the main effect of Phase provided the best account of the data, and was preferred over models additionally including Condition, *BF* = 4.35, or the Phase × Condition interaction, *BF* = 16.39, reflecting the differing time scales of the encoding and recognition responses. Critically, the inclusion Bayes factor for Condition was *BF*_incl = 0.194 (*BF*_excl ≈ 5.15), and for the Phase × Condition interaction *BF*_incl = 0.199 (*BF*_excl ≈ 5.03), indicating moderate evidence *against* including either term. Descriptively, RTs were comparable for abstract and concrete trials both during encoding (*M*_abstract = 20.017 s, *SD* = 6.219; *M*_concrete = 19.661 s, *SD* = 6.500) and during recognition (*M*_abstract = 3.487 s, *SD* = 3.827; *M*_concrete = 3.601 s, *SD* = 5.026). Together, these results provide moderate evidence against a difference in reaction time between concrete and abstract trials at either phase, indicating that the enhanced recognition of faces following abstract-concept conversations is unlikely to be explained by greater cognitive effort or more elaborate processing of abstract material during encoding or recognition.

## Discussion

The present study investigated whether the type of concept discussed in a text-based, computer-mediated interaction modulates memory for interlocutors’ social identities. Recognition memory for interlocutors’ faces, but not their names, was enhanced after conversations about abstract concepts relative to conversations about concrete concepts. These results are consistent with the Words as Social Tools (WAT) framework (Borghi and Binkofski 2014; Borghi et al. 2019), according to which the grounding of abstract concepts recruits social-cognitive resources more extensively than the grounding of concrete concepts, specifically, through heightened reliance on social metacognition during interaction. Although these processes were not directly operationalized in the present study, they provide a theoretical framework for interpreting the observed effects.

The study employed a two-component paradigm adapted from Villani et al. (2022): participants first engaged in a conversational phase responding to abstract and concrete concept prompts from virtual agents, which served as the encoding phase, and subsequently completed a recognition task for those agents’ names and faces, along with a measure of perceived closeness. Analyses of the conversational phase confirmed that abstract concept discussions elicited the expected interactional signatures: more uncertainty expressions, questions, turn-taking, general statements, and affective and belief-related language (H1-H4), replicating Villani et al. (2022) in a Romanian-speaking sample. These conversational dynamics served as an embedded manipulation check, establishing that encoding conditions differed in the theoretically predicted direction and providing a principled basis for interpreting the memory and closeness results that follow (H5-H7).

### Conversational dynamics

Consistent with Villani et al. (2022), abstract concepts (ACs) elicited more uncertainty expressions, more questions, increased turn-taking, and a higher frequency of general statements than concrete concepts (CCs), as reflected in the corresponding linguistic markers. The only effects for which the credible intervals included zero were those for the number of questions and repetitions of the target word. Overall, these findings largely support the adequacy of applying the Italian operationalization of abstractness and concreteness to the Romanian language in the context of the present study.

ACs lack clear, directly accessible referents in the environment and are therefore more difficult to communicate and understand. They are more dependent on experience and context (Schwanenflugel et al. 1992) and are also more contestable (Gandolfi et al. 2023). This provides a parsimonious explanation for the effects observed in our language production task: participants were more uncertain about the intended meanings of ACs than of CCs, which in turn elicited different communicative strategies. Uncertainty expressions function as signals to interlocutors that additional support may be needed. These signals do not necessarily constitute explicit requests for help, such as direct questions or turn-taking. However, in social interactions, where individuals are motivated to reduce uncertainty (Berger and Calabrese 1975), they nonetheless serve as meaningful indicators of uncertainty management.

Participants asked for more cues when attempting to interpret abstract concepts than when processing concrete concepts in experimental tasks (Fini et al. 2021), an effect that was replicated in the present study. Assigning a turn to the interlocutor often coincided with the use of a question, but participants also employed other strategies, such as requests for elaboration or expressions of interest in receiving further input. In this way, participants deferred to others to provide additional information about the meaning of the message or the contextual details grounding the abstract concepts being communicated. They also produced more uncertainty expressions in the AC condition. These behaviors should not be interpreted merely as markers of confusion, but rather as metacognitive signals that facilitate coordination within what Pickering and Garrod (2021a, b) have termed the “shared workspace of dialogue”. Such signals can be contrasted with strategies that discourage further contributions to the interaction by creating the appearance that conversational alignment has already been achieved. One example is the use of general statements, whereby an agent refrains from engaging further in the conversation and instead offers a broad remark that may signal understanding even when such understanding has not in fact been established.

Judging one’s own mental states as inadequate until proven otherwise may constitute a form of socially adaptive metacognitive self-monitoring that prompts individuals to track both their internal states and the conversational context (Shea 2018). This mechanism may account for the observed associations among conversational features at the subtype level. Nevertheless, we also identified divergent effects across subtypes.

Physical Spatial-Temporal Quantitative concepts elicited more uncertainty expressions and general statements than any other abstract subtypes, a pattern that is not fully consistent with previous findings. In earlier work, the Philosophical-Spiritual concept domain (typically regarded as the most prototypically abstract) was identified as the most uncertain, as indexed by multiple features (Villani et al. 2022). In the present study, this domain elicited the highest levels of turn-taking and the greatest number of questions. However, the Physical Spatial-Temporal Quantitative subtype exhibited more abstract-like behavior in terms of uncertainty expressions and general statements. Words as Social Tools theory proposes that different cultures, precisely because of the central role of social and linguistic experience, ground concepts in distinct ways (Borghi and Cimatti 2009; Borghi and Binkofski 2014; Borghi et al. 2019; Mazzuca et al. 2025a). This cultural variability was anticipated and motivated our decision to begin with a preliminary, context-sensitive validation of abstractness and concreteness.

This unexpected pattern may reflect at least two non-mutually exclusive processes. One straightforward account is that Physical-Spatio-Temporal-Quantitative concepts are perceived as more objective, factual, or conventionally grounded than Emotional-Social or Philosophical-Spiritual concepts, requiring less meaning negotiation and naturally eliciting consensus-based rather than exploratory statements. A second, cultural interpretation is also possible: abstractness may be represented differently across cultural contexts, and Romania, which shows the highest share of negative views on science among European countries (European Commission 2024), and where conservative ideologies and lower scientific literacy have been associated with distrust in science (Obreja et al. 2025; Alper et al. 2024) may diverge from the Italian context in this respect. Given that participants in both the present study and Villani et al. (2022) were university students, a simple lack-of-knowledge account is less plausible; the use of general statements may instead reflect a form of discursive disengagement shaped by sociopolitical context. Both interpretations are speculative and cannot be distinguished with the current design; we therefore encourage future research to disentangle them through targeted cross-cultural norming and replication.

Beyond uncertainty-related features, additional support for the adequacy of applying the Italian abstract–concrete distinction to Romanian comes from internal situational features, which were more prevalent in the AC condition. The strongest evidence emerged for the affective dimension: ACs elicited more emotional content, as indicated by a higher frequency of emotion-denoting words. The role of emotions in grounding abstract concepts has been emphasized in previous research (Kousta et al. 2011). As expected, the Emotional Mental States and Social subtype of ACs yielded the highest proportion of explicitly expressed affective states. These robust preliminary findings can also be interpreted in light of converging evidence showing that affective concepts are processed faster and recalled better than CCs, yet differently from other ACs; they elicit distinct relational patterns and are acquired earlier than other abstract concepts (Barca et al. 2017; Ponari et al. 2018). Accordingly, affective concepts may be regarded as a distinct category that cuts across the traditional abstract–concrete dichotomy.

AC sentences elicited a higher frequency of explicitly expressed beliefs than CC sentences. The observed small-to-moderate effect was robust and supports a core tenet of the Words as Social Tools theory (Borghi 2013; Borghi and Binkofski 2014; Borghi and Cimatti 2009), namely the central role of social and linguistic input in the acquisition and representation of abstract concepts. Overt belief expressions serve an inherently social function by explicitly communicating one’s interpretations and intended meanings to others. In this sense, they constitute a form of social metacognition, insofar as they acknowledge the potential for divergent interpretations across interlocutors and facilitate alignment. Among ACs, Emotional Mental States and Sociality concepts elicited the highest frequency of overt belief expressions, a pattern that is consistent with this dynamic, hybrid grounding account.

By contrast, evidence for effects related to metacognition and introspection was insubstantial. Although prior work has suggested links between ACs, introspection, and social metacognition (Harris and Koenig 2006; Mazzuca and Santarelli 2022), the study on which our preliminary validation was based reported similar null or weak effects for these dimensions (Villani et al. 2022). Taken together, the pattern across all nine coded features provides initial evidence that the concepts employed in the present study were appropriately adapted from Italian to Romanian within our experimental procedure. Because these interactional features occurred during the same conversational exchanges that served as the encoding phase for subsequent recognition, they provide a principled basis for interpreting differences in social memory as downstream consequences of concept-type-specific processing demands.

### Selective enhancement of face recognition for abstract concepts

Starting from the assumption that a social stimulus becomes more salient through the social metacognitive processes of storage and manipulation, both core components of social cognition, the primary aim of this study was to examine whether prior engagement in discussions involving abstract concepts would enhance subsequent recognition of, and perceived closeness to, an interlocutor’s name and face relative to discussions of concrete concepts. The present study introduced a reproducible experimental paradigm to examine whether discussing abstract versus concrete concepts modulates memory for interlocutors’ social identities and willingness to continue the interaction. Faces associated with abstract-concept trials were recognized more accurately than faces associated with concrete-concept trials, whereas this effect did not extend to names. The results provide initial support for the utility of this procedure and for a link between social interaction, social cognition, and concept-type–dependent processing.

We observed a robust difference in recognition memory for faces, with more faces recognized in the AC condition than in the CC condition. This effect did not extend to names. Our contention is that this is related to the privileged, highly informative position of face stimuli among social cues: they are processed more rapidly than names (Moret-Tatay et al. 2021), and they are evaluated automatically for trustworthiness, competence, and emotional state, even at minimal exposure (Todorov et al. 2009; Todorov 2012; Mason et al. 2006). Faces convey, moreover, cues about competence, group membership (Hong et al. 2023), stereotypes (Mason et al. 2006), social categories, and personality traits (Todorov 2012). Crucially, these evaluations are not passive: they are socially motivated, driven by the perceiver’s need to assess whether the interlocutor is a reliable and relevant epistemic source (Ghuman et al. 2014). The higher recognition accuracy for faces associated with agents from the abstract condition, then, is explained by the process inherent in the social grounding of abstract concepts, where faces are needed to assess others and what they want to communicate. This interpretation is consistent with evidence that person recognition integrates faces and voices, both high-information social signals, rather than faces and names (O’Mahony and Newell 2012).

### Null effects for name recognition and perceived closeness

The models run for perceived closeness yielded insubstantial evidence, in line with the findings of Fini et al. (2023), who reported a numerical trend that was not subsequently replicated. We operationalized closeness differently from Fini et al., using a task-based measure rather than an interpersonal distance task or an Inclusion of Other in the Self scale. Specifically, we assessed participants’ willingness to continue the conversation in a face-to-face setting, which we considered more contextually appropriate than alternative measures. We deliberately avoided distinguishing between psychological and physical proximity, as this distinction was not theoretically necessary for our aims. Accordingly, our measure integrated both dimensions, under the assumption that participants would be unlikely to choose an in-person interaction in the absence of a positive and sufficiently clear impression, given that such a setting implies not only physical proximity but also increased spontaneity and social exposure.

The social processing of abstract concepts may therefore not directly influence the evaluation of specific agents as more suitable to rely on, even though conversational interaction typically aims at achieving alignment. In other words, alignment can be reached and mutual understanding achieved without necessarily giving rise to attachment or perceived closeness. Nonetheless, specific social stimuli (faces, but not names in the present study) may become more salient in memory following engagement in discussions involving abstract concepts. Several explanations for this dissociation can be proposed. The present methodology, particularly in light of the supplementary analyses, allows us both to adjudicate among them and to generate testable hypotheses for future research.

erhaps the greater cognitive load or processing difficulty associated with abstract concepts compared to concrete ones might be the most convincing alternative explanation. On this account, interlocutors from the abstract condition may have been better recognized because responding to abstract concepts required greater effort or arousal, which in turn enhanced overall recognition memory for that condition. However, increased cognitive load is typically associated with poorer incidental memory, particularly for task-irrelevant stimuli, which makes this explanation unlikely (Lavie 2005). In the present task, neither the interlocutor’s name nor profile picture was directly relevant to participants’ response requirements. Moreover, if cognitive load were the driving factor, comparable effects would be expected across different types of social stimuli. The absence of a corresponding effect for names further challenges this interpretation.

As the second supplementary analysis shows, there was no increased time-on-task between the abstract and the concrete conditions, neither in the encoding nor in the recognition phase. This means that the higher recognition accuracy for faces associated with abstract-concept conversations cannot straightforwardly be attributed to greater cognitive effort or slower, more elaborate processing during the abstract condition. This pattern is also not easily accommodated by a purely domain-general resource account, since names are, if anything, typically recognized at least as well as faces in matched recognition paradigms (Burton et al. 2019), making it unlikely that the dissociation reflects a generic allocation of resources toward “more effortful” material. The overall face advantage observed in our study, opposite to Burton et al. (2019), likely reflects the concurrent verbal demands of composing a conversational reply, which competed with name phonological encoding while leaving the largely automatic, perceptually-driven encoding of faces comparatively unaffected. It explains why names underperformed faces overall, but not why the abstract-concept manipulation selectively enhanced face memory, which remains inconsistent with a simple domain-general effort account.

Against this background, the present findings are particularly informative because they indicate that the selective advantage for faces following abstract-concept discussions is unlikely to be explained solely by differences in time-on-task or by a general superiority of facial stimuli in memory. Instead, the results suggest a more specific interaction between abstract-concept processing and the social-cognitive mechanisms involved in person recognition, consistent with the Words as Social Tools theory (Borghi and Binkofski 2014; Borghi et al. 2019). The selective enhancement of memory for faces, without a corresponding advantage for names or other associated information, may reflect greater engagement in evaluating and monitoring the interlocutor during abstract-concept discussions.

### Limitations and future directions

Our study has several limitations indicating that these results require further investigation and refinement. First, future studies could rely on abstract and concrete concepts drawn from established norming or feature-elicitation studies that psycholinguistically validate this distinction.

Second, the communicative context of the present study was deliberately minimal: participants produced a single-turn response to a scripted prompt. They did not receive individualized replies, which may appear artificial as a conversational opener. While this design allows tight experimental control over concept type, ecological validity could be increased by moving to a more bidirectional, flexible, context-sensitive, multi-turn interaction. Such a design, however, would also impose greater demands on participants (e.g., increased time commitment) and could introduce additional confounding variables, such as the inclusion of multiple concepts within the same conversational exchange. This asymmetric, non-reciprocal structure is also relevant to the null effect for perceived closeness, which warrants particular caution in interpretation. Unlike the conversational paradigm in Fini et al. (2023), which involved genuine bidirectional exchanges, the present task was structurally asymmetric: participants responded to pre-scripted prompts from virtual agents and did not receive tailored responses. Interpersonal closeness, as theorized in Uncertainty Reduction Theory (Berger and Calabrese 1975), typically emerges from mutual self-disclosure and reciprocal exchange, conditions that were not met in the present design. It is therefore possible that the absence of real-time, bidirectional engagement suppressed closeness development regardless of concept type, creating a floor effect. Future research should examine whether the face-recognition advantage for abstract concepts, and any associated effects on closeness, are amplified in genuinely interactive dyadic conversations. Across multiple turns, an interlocutor’s contributions, for example, agreement or disagreement on a contested concept, may reveal their values, expertise, or group memberships and thereby progressively enrich the participant’s representation of that person. Such meaning-relevant updating may make some interlocutors more memorable than others and allow the social-grounding demands of abstract concepts to be tested more sensitively.

Third, we did not include an independent baseline measure of non-social memory ability. Thus, although the within-participant design and the dissociation between name and face recognition argue against a simple general-memory explanation, future studies should include baseline memory measures to better isolate the contribution of concept type to social recognition. Relatedly, from an analytical perspective, although Bayesian analyses can be informative with relatively modest sample sizes, our sample may be considered small for detecting a robust social effect on memory as mediated by abstract concept processing. Future studies could therefore aim to replicate these findings with larger samples or build cumulatively on the present data in order to assess the stability of the observed effects.

Finally, although the face stimuli were not selected specifically to be matched on perceived trustworthiness, normative ratings from the Chicago Face Database indicated that the faces used in the study were rated close to neutral and showed relatively limited variability on this dimension (M = 3.41, SD = 0.35, on a 1–7 scale). This reduces, but does not eliminate, the possibility that perceived trustworthiness influenced recognition performance, particularly because trustworthiness was not formally balanced across experimental conditions or included as an item-level predictor. Future studies should therefore either match face stimuli on normative trustworthiness ratings or model these ratings directly when examining recognition outcomes. We also did not examine the correspondence between participants’ gender and the gender of the depicted interlocutors. Gender congruence or incongruence may moderate both face-recognition memory and willingness to continue the interaction and should therefore be examined in future research.

## Conclusion

The present study makes three main contributions. First, it demonstrates that discussing abstract concepts in a text-based, computer-mediated interaction selectively enhances recognition memory for interlocutors’ faces, but not their names, a distinction that is not easily explained by general cognitive load or time-on-task accounts. Second, this face-salience effect provides novel empirical support for the Words as Social Tools (WAT) framework: when conceptual meaning is uncertain and requires social grounding, the epistemic significance of interlocutors is heightened, prioritizing the encoding of socially informative cues such as faces. Third, the study introduces a methodologically tractable, cost-effective paradigm that successfully replicates the Italian conversational dynamics findings in a Romanian-speaking population, extending the cross-linguistic scope of the WAT framework. Future research should examine these effects with larger samples, genuinely interactive designs, and culturally diverse populations.

## Data Availability

The datasets, analysis scripts, and supplementary materials supporting this study are available on the Open Science Framework: https://osf.io/9hqcy/overview?view_only=3b3852b4d9804533a3ada3ad92e4a535.
